# Daily time series of 12 human thermal stress indices in Greece, aggregated at commune level (1998–2022)

**DOI:** 10.1016/j.dib.2024.111264

**Published:** 2024-12-28

**Authors:** Georgios Charvalis, Michalis Koureas, Chloe Brimicombe, Chara Bogogiannidou, Fani Kalala, Varbara Mouchtouri, Christos Hadjichristodoulou, for HIGH Horizons Study Group

**Affiliations:** a Laboratory of Hygiene and Epidemiology, Faculty of Medicine, University of Thessaly, 41222 Larissa, Greece; b Department of Immunology and Histocompatibility, Faculty of Medicine, University of Thessaly, 41500 Larissa, Greece; c Wegener Center for Climate and Global change, University of Graz, Brandhofgasse 5, 8010 Graz, Austria

**Keywords:** Heat, Thermal discomfort, Temperature, ERA5-land, ERA5, Heatwave, Climate change, Spatial aggregation

## Abstract

In this paper we present a dataset that contains daily mean, maximum and minimum values of 12 heat stress indices averaged over Greek communes from January 1998 to December 2022. The heat indices contained in the dataset include Apparent Temperature (AT), Heat Index (HI), Humidity Index (Humidex), Normal Effective Temperature (NET), Wet Bulb Globe Temperature (simple version WBGT), Wet Bulb Globe Temperature (thermofeelWBGT), Wet Bulb Temperature (WBT), Wind Chill Temperature (WCT), Mean Radiant Temperature (MRT), and Universal Thermal Climate Index (UTCI) with two variations (UTCI indoor and UTCI outdoor).

To develop the dataset, we used hourly climate variables, acquired from the ERA5 and ERA5-Land datasets, produced by the European Centre for Medium-Range Weather Forecasts (ECMWF), which are accessible through the Copernicus Climate Change Service (C3S) Climate Data Store (CDS) Application Program Interface (API) client. We used freely available python scripts and resources (HiTiSEA repository, thermofeel library), to calculate 12 heat stress indices for Greece at an enhanced spatial resolution of 0.1° × 0.1°. To facilitate geospatial analysis over the Greek communes, boundary data in shapefile format were obtained from the Hellenic Statistical Authority (ELSTAT). The execution of a built-in QGIS function was implemented to geospatially aggregate the NetCDF files of 12 daily mean, maximum and minimum, indices to 326 Greek communes for 9131 days.

The high spatial and temporal resolution of the data, makes the dataset appropriate for analysis and comparison of climate change impacts, heatwave patterns, and the development of climate adaptation strategies at a regional scale in Greece. Additionally, it can be used as a basis of a system to inform and devise targeted interventions and policies aimed at mitigating the effects of extreme heat events. The attribution of heat stress indices at the commune level (also referred as municipalities or municipal units), which is the lowest level of government within the organizational structure in Greece, enhances the usefulness of the data for statistical analysis against other parameters, such as epidemiological or socio-economic data, which are often available at this level. Finally, the dataset can support educational purposes, providing a practical example of climate data analysis and geospatial statistics applications.

Specifications Table

**Table:** 

Subject	Climatology, Public Health and Health Policy
Specific subject area	Thermal stress indices are quantitative composite measures used to evaluate and assess the combined effects of climatic factors (temperature, humidity, wind speed, radiation) in humans. These indices aim to provide a comprehensive assessment of thermal comfort and potential heat-related stress. Each index has a specific focus and methodology, allowing for a detailed analysis of different aspects of thermal stress.
Type of data	Daily time series of 12 thermal stress indices of zonal statistics averaged across Greek communes by statistical measure (mean, max, min) in comma-separated text table files (.csv) Vector file consisting of Greek communes (.shp and associated files) Python script text files (.py)
Data collection	Raw data were acquired via the C3S Climate Data Store API client. Specific hourly climatic variables (in UTC+00:00 time reference) of the ERA5-Land [1] and ERA5 [2] reanalysis datasets were downloaded for the spatial and temporal extent. Python scripts from the HitiSEA [3] repository were used to process and save raw data in NetCDF format for a bounding box over Greece, extending 19°E to 29°E and 35°N to 42°N. QGIS (version 3.28.7-Firenze) python console was used for zonal statistics analysis. Other resources include the ECMWF thermofeel library [4], Python scripts for calculations and data manipulation, and geospatial commune boundaries in shapefile format, downloaded from the Hellenic Statistical Authority.
Data source location	Primary data sources: • ERA5-Land dataset and ERA5 reanalysis datasets via Copernicus Climate Data Store API client (https://cds.climate.copernicus.eu/)
	• Greek commune geospatial boundary data from the Hellenic Statistical Authority (ELSTAT) https://www.statistics.gr/documents/20181/1194366/kallikratikoi_dimoi.rar/ea9336e4-ee2d-45b7-b74d-b4f63f99b12e
	All calculations were executed and stored on a personal computer with Windows 11 Pro Version 23H2 operating system.
Data accessibility	The dataset and its metadata are freely available without registration in the Zenodo repository with DOI: 10.5281/zenodo.10955209 for research purposes. Direct URL to data: https://zenodo.org/uploads/10955209 Direct URL to data for un-authenticated users: https://zenodo.org/records/10955209?preview=1&token=eyJhbGciOiJIUzUxMiJ9.eyJpZCI6IjgxNzk5NDJkLWQ4NzItNDcwYi1hZDc3LTNiM2I0NWQxZDYwMiIsImRhdGEiOnt9LCJyYW5kb20iOiI4YzllOWU1M2ZlMDk2ZWY2NGYyMzM0MjgzY2IyMjNlMSJ9.MCfcmenQDdnnZBcY6M4TZXdLjApkgvvqxZ4OY97JSlAznQ4s3_2ILFSpdSfHOEPUqbt29Jv02RHe-1CvJnq2aQ
Related research article	not applicable

## 1 Value of the Data

• Provision of time series data on various human thermal stress indices provides a comprehensive understanding of thermal conditions. These indices are crucial for assessing human thermal stress, which has direct implications for public health, especially during extreme weather events like heatwaves.
• This dataset addresses the problem that most heat stress indices are not currently available in the ERA5-Land dataset resolution of 0.1° × 0.1°, which allows for more detailed analysis compared to ERA5′s coarser resolution. This is particularly useful in capturing microclimates and small-scale geographic variations.
• The dataset spans from January 1, 1998, to December 31, 2022, offering a long-term perspective on climate trends and variations. The dataset's extensive temporal range supports long-term climate studies, allowing researchers to analyze trends, variability, and correlations of the heat indicators with various social and environmental variables over a significant period.
• Attribution of heat stress indices at commune level enables the direct comparison and merging with other types of data such as epidemiological and socioeconomic data that are often expressed at commune level.
• Repository contains data in CSV format ensuring broad compatibility and ease of use, making it accessible to researchers across various software and languages. Thus, facilitating straightforward data processing and analysis.
• Freely available Python scripts and resources are shared to allow users to reproduce the methodology for any part of the world.

## 2 Background

The increasing frequency and intensity of heatwaves due to climate change has amplified the need for detailed studies on thermal stress and its impacts on human health. Greece has a diverse geography and climate, providing a valuable case for examining potential effects on public health, tourism and other human activities. To address this, we have compiled a comprehensive dataset, HTSI-GR, encompassing a daily time series of 12 thermal stress indices averaged over Greek communes from January 1998 to December 2022 [5].

## 3 Data Description

According to the overview table of the Human Thermal Stress Indices (HTSI-GR) dataset (Table 1), the repository containing the heat indices is organised into 36 CSV files, each named according to the specific index, the statistical measure, and the date range covered in the yyyy-mm-dd format. The naming convention for the files follows the format: <Heat Index Name>_<statistical measure>_<Start Date>_<End Date>.csv

**Table 1:** The overview table of the HTSI-GR dataset.

Heat indices names	Description	Units	Source reference	Implementation methodology reference	Dataset file names
AT	Apparent Temperature	°C	[6]	[3]	AT_min_1998-01-01_2022-12-31.csv, AT_mean_1998-01-01_2022-12-31.csv, AT_max_1998-01-01_2022-12-31.csv
HI	Heat Index	°C	[7]	[3]	HI_min_1998-01-01_2022-12-31.csv, HI_mean_1998-01-01_2022-12-31.csv, HI_max_1998-01-01_2022-12-31.csv
Humidex	Humidity Index	°C	[8]	[3]	Humidex_min_1998-01-01_2022-12-31.csv, Humidex_mean_1998-01-01_2022-12-31.csv, Humidex_max_1998-01-01_2022-12-31.csv
NET	Normal Effective Temperature	°C	[9]	[3]	NET_min_1998-01-01_2022-12-31.csv, NET_mean_1998-01-01_2022-12-31.csv, NET_max_1998-01-01_2022-12-31.csv
WBGT	Wet Bulb Globe Temperature (simple version)	°C	[10]	[3]	WBGT_min_1998-01-01_2022-12-31.csv, WBGT_mean_1998-01-01_2022-12-31.csv, WBGT_max_1998-01-01_2022-12-31.csv
thermofeelWBGT	Wet Bulb Globe Temperature	°C	[11]	[4]	thermofeelWBGT_min_1998-01-01_2022-12-31.csv, thermofeelWBGT_mean_1998-01-01_2022-12-31.csv, thermofeelWBGT_max_1998-01-01_2022-12-31.csv
WBT	Wet Bulb Temperature	°C	[11]	[3]	WBT_min_1998-01-01_2022-12-31.csv, WBT_mean_1998-01-01_2022-12-31.csv, WBT_max_1998-01-01_2022-12-31.csv
WCT	Wind Chill Temperature	°C	[12]	[3]	WCT_min_1998-01-01_2022-12-31.csv, WCT_mean_1998-01-01_2022-12-31.csv, WCT_max_1998-01-01_2022-12-31.csv
MRT	Mean Radiant Temperature	°C	[13]	[3]	MRT_min_1998-01-01_2022-12-31.csv, MRT_mean_1998-01-01_2022-12-31.csv, MRT_max_1998-01-01_2022-12-31.csv
UTCI	Universal Thermal Climate Index (UTCI)	°C	[14]	[3]	UTCI_min_1998-01-01_2022-12-31.csv, UTCI_mean_1998-01-01_2022-12-31.csv, UTCI_max_1998-01-01_2022-12-31.csv
UTCI2	Indoor environment UTCI with 2 parameters (air temperature and humidity)	°C	[14]	[3]	UTCI2_min_1998-01-01_2022-12-31.csv, UTCI2_mean_1998-01-01_2022-12-31.csv, UTCI2_max_1998-01-01_2022-12-31.csv
UTCI3	Outdoor shaded space environment UTCI with 3 parameters (air temperature, humidity, and wind speed)	°C	[14]	[3]	UTCI3_min_1998-01-01_2022-12-31.csv, UTCI3_mean_1998-01-01_2022-12-31.csv, UTCI3_max_1998-01-01_2022-12-31.csv

The contents of the CSV files are structured as follows: the first column (“KALCODE”) contains 326 unique identifier codes for the communes, used by the Hellenic statistical service, while the second (“LEKTIKO”) includes the official Greek names of the communes. The subsequent 9131 columns are named with dates followed by the specific heat index name and statistical measure, for example, 1998-01-01_UTCI_mean, 1998-01-02_UTCI_mean, …, 2022-12-31_UTCI_mean.

The repository also contains a folder with additional resources containing scripts to replicate the results, other necessary files, and replication instructions. A brief description of the contents of the folder is available in the table below (Table 2).

**Table 2:** The overview table of the additional resources folder provided in the HTSI-GR dataset repository.

File names	Description
0.Calculate thermofeelWBGT.py	A python script that calculates the Wet Bulb Globe Temperature (WBGT) using the Thermofeel library. Processes NetCDF files containing daily meteorological data and outputs WBGT values in new NetCDF files for each day.
1.Merge_HI_by_max-mean-min.py	A python script that merges daily NetCDF files containing heat index (HI) data into three separate files based on mean, maximum and minimum values for further processing.
2.QGIS_zonal_statistics.py	A python script that calculates zonal statistics for heat indices using QGIS python console. Uses a shapefile of Greek communes and a raster NetCDF file containing daily index values, and outputs daily CSV files with computed statistics.
3.Zonal_format.py	A python script that formats the zonal statistics results into a comprehensive dataset. Combines daily CSV files into a single CSV, fills in missing data using nearest neighbour values, and produces a final formatted dataset.
Greek Communes.ZIP	Contains the shapefile of Greek communes derived from the Hellenic Statistical Authority (ELSTAT) required for zonal statistics calculations.
Nearest Neighbour data table.csv	A support table to script 3.Zonal_format.py that lists communes with missing data and their nearest neighbour with data.
Read me.txt	Provides an overview and instructions for using the scripts. Describes the purpose of each script, lists prerequisites, and provides step-by-step instructions for replicating the dataset.

## 4 Experimental Design, Materials and Methods

**Fig. 1:**
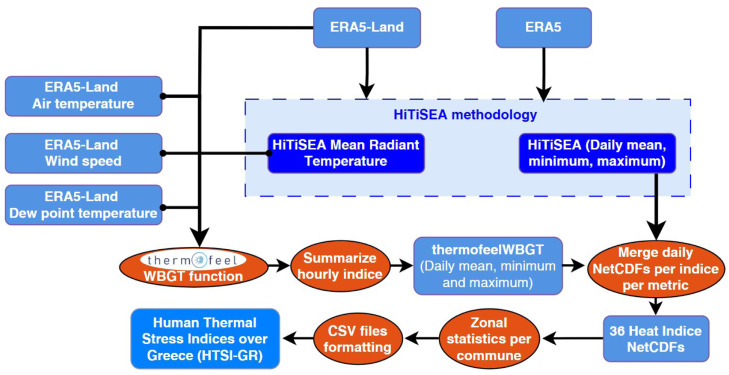
The workflow implemented to create the dataset from ERA5-Land and ERA5 climate reanalysis variables.

**Fig. 2:**
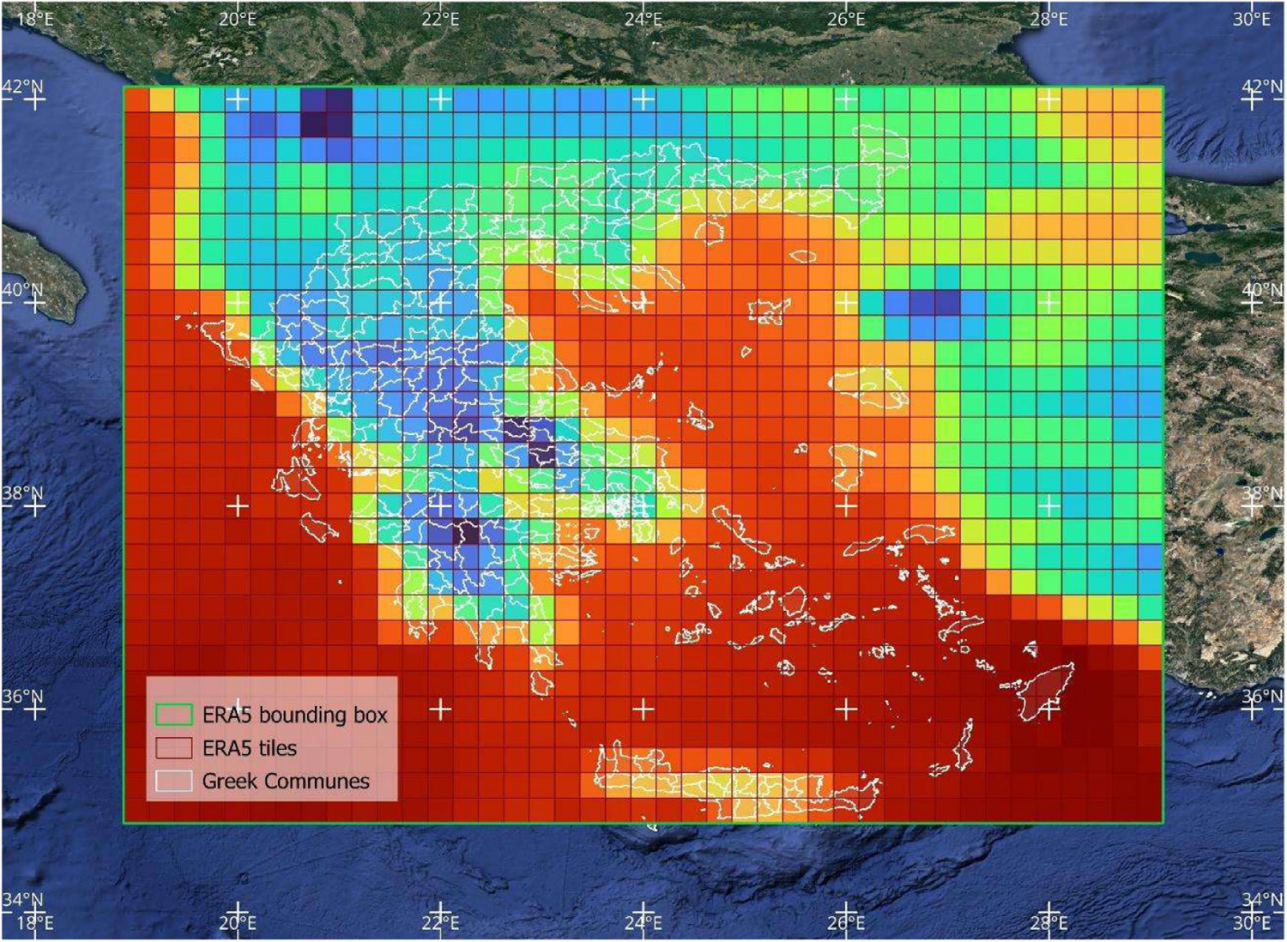
ERA5 0.25° × 0.25° tiles. ERA5 bounding box used, and Greek communes administrative boundaries are visible. ERA5 data tiles are colored based on the daily mean temperatures of 1-1-2020

**Fig. 3:**
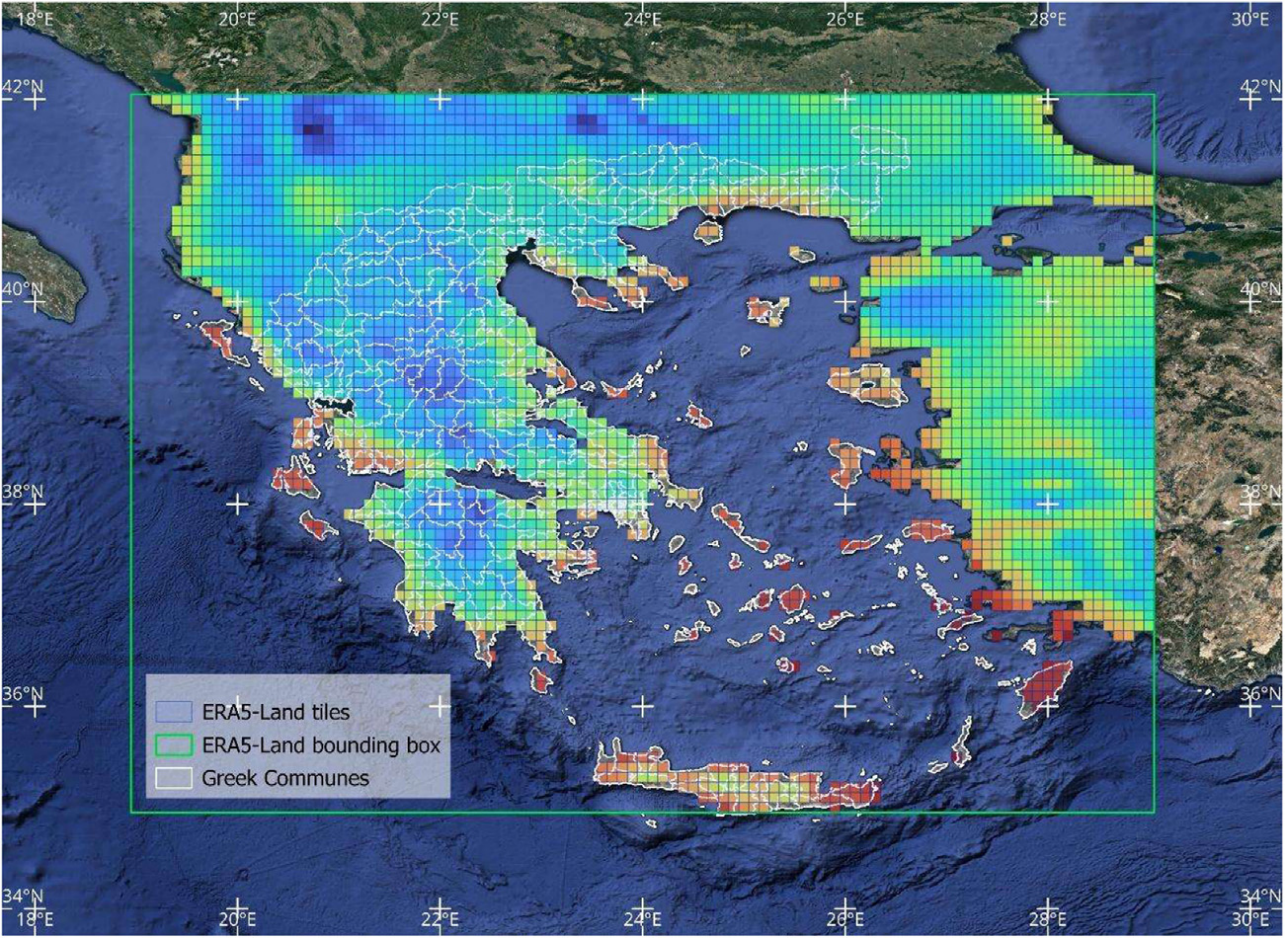
ERA5-Land 0.1° × 0.1° tiles, ERA5-Land bounding box used, and Greek communes administrative boundaries are visible. ERA5-Land data tiles are colored based on the daily mean temperatures of 1-1-2020.

As depicted in Fig. 1, the first part of the workflow calculates eleven heat indices at a spatial resolution of 0.1° × 0.1° based on the methodological approach followed by Yan et al. [3]. The authors provide python scripts to replicate the eleven HiTiSEA indices over any part of the globe within valid ERA5-Land and ERA5 tiles. Environmental Stress Index (ESI) was excluded from the analysis due to high noise levels and the presence of extreme values. The download boundaries in the script using the CDS API client were defined as 19°E to 29°E and 35°N to 42°N for the needs of the present dataset. Hourly raw data are calculated in UTC+00:00. An illustration of the area of interest, the input data tilling and commune boundaries are available in Fig. 2, Fig. 3.

The workflow utilizes only one ERA5 variable: the Total Sky Direct Solar Radiation at Surface (fdir), which is used to approximate direct solar radiation at the surface in Joules per square meter (J/m²). The downsampling from the 0.25° × 0.25° to 0.1° × 0.1° spatial resolution was executed by using nearest-neighbour interpolation, to match the resolution of ERA5-Land variables. Climatic variables from ERA5-Land used in the workflow include:

• Air Temperature (t2m): 2-meter temperature in Kelvin (K).
• Dew Point Temperature (d2m): 2-meter dew point temperature in Kelvin (K).
• Wind Speed Components (u10 and v10): 10-meter U and V wind components in meters per second (m/s).
• Surface Pressure (sp): Atmospheric pressure at the surface in Pascals (Pa).
• Surface Net Short-Wave Radiation (ssr): Net solar radiation at the surface in Joules per square meter (J/m²).
• Surface Short-Wave Radiation Downwards (ssrd): Downward flux of solar radiation at the surface in Joules per square meter (J/m²).
• Surface Net Long-Wave Radiation (str): Net thermal radiation at the surface in Joules per square meter (J/m²).
• Surface Long-Wave Radiation Downwards (strd): Downward flux of thermal radiation at the surface in Joules per square meter (J/m²).

**Fig. 4:**
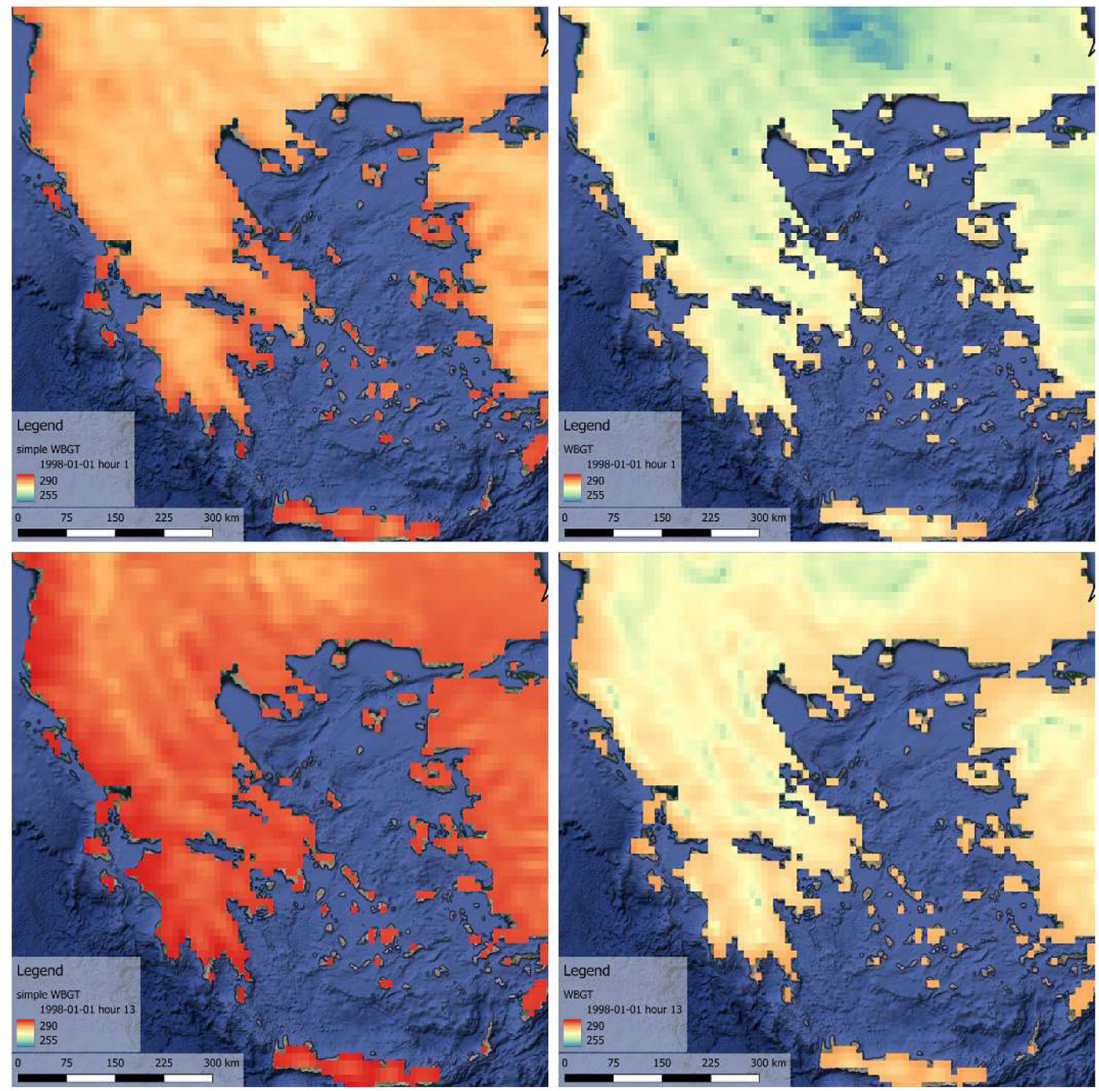
A visual comparison of WBGT simple [10] (left) and WBGT derived from thermofeel library [4] (right). Each method is presented with two instances. The top represents midnight conditions, and the bottom represents noon conditions. The colour palettes are comparable in all four instances.

After we executed all HiTiSEA python scripts (available in the CalcHiTiSea_pkg folder of the respective repository: https://doi.org/10.6084/m9.figshare.c.5196296) we used the hourly Mean Radiant Temperature produced, and three ERA5-Land variables to call the calculate_WBGT function provided by the thermofeel library [4]. The rationale behind adding the WBGT index by using the thermofeel library is that the existing WBGT index produced by the HiTiSEA, uses the simple version of the equation as described in [10]. This model, which relies solely on air temperature and humidity, has been criticised for providing an incomplete representation of WBGT since it fails to account for the effects of global temperature [15]. The simple version produced oversaturated results in comparison with the WBGT produced by using the thermofeel library [4,12] (available at https://github.com/ecmwf/thermofeel), which can adapt more dynamically to local variances as depicted in the figure below (Fig. 4).

Hourly values of the thermofeel WBGT were then summarised to match the daily formatting of the rest of the indices and then were all concatenated for the time span to finally create one file per available index per statistical measure (36 different NetCDFs) for easier manipulation. Final step was to calculate averaged zonal statistics per Greek commune on the QGIS (version 3.28.7-Firenze) python console by calling the native:zonalstatisticsfb function with the selected heat index NetCDF file and the shapefile of the Greek communes as arguments. Finally, a last formatting step was performed on the zonal statistics results. Communes with missing data due to ERA-5 Land tiling (a few small islands and coastal communes) were assigned their nearest neighbours heat index values by using the file "Nearest Neighbour data table.csv" that links communes with no data with their nearest neighbour commune with data, to produce the final formatted CSV file.

## Limitations

The intra-commune heterogeneity of the terrain and landscape features is not accounted for in this dataset. It assumes uniform thermal conditions across each commune due to the zonal statistics averaging method. Localized effects, such as urban heat island effect, are likely inefficient to be addressed with this dataset especially for larger communes. Lastly, the produced indices have not been validated against historical data from weather stations within the communes yet.

## Ethics Statement

The authors have read and follow the ethical requirements for publication in Data in Brief and confirm that the current work does not involve human subjects, animal experiments, or any data collected from social media platforms.

## Credit Author Statement

**Georgios Charvalis:** Methodology, Writing, Software, Data Curation, **Michalis Koureas:** Conceptualization, Writing and Reviewing, **Chloe Brimicombe:** Methodology, Writing - Review & Editing, **Chara Bogogiannidou:** Reviewing and Editing, Writing - Original Draft, **Fani Kalala:** Supervision, Writing - Review & Editing, **Varbara Mouchtouri:** Supervision, Project administration, **Christos Hadjichristodoulou:** Supervision, Conceptualization.

## Data Availability

ZenodoDaily time series of 12 human thermal stress indices in Greece aggregated at commune level (1998-2022) (Original data).

## Appendix Supplementary materials

Image, application 1
